# Crystal Gold for Filling Teeth

**Published:** 1854-01

**Authors:** 


### Editorial.

### CRYSTAL GOLD FOR FILLING TEETH.

It appears that there is ever to be something new in the profession, and, as this is an age of progress, why should not dental science be in a progressive developement? Men of education and literary attainment ask us repeatedly, "if the Profession is in a progressive state?" We answer, most assuredly, and although some of her *stakes* may be defective and give way beneath the mass of truth and error which is being accumulated, yet truth is elastic, and will bound from the earth, and as she rises shake off the error which obscured her brightness, and like the sun, burst forth from the cloud which obscured her rays with increased refulgence. We have great faith in this progress, and regard every step in the developement of those principless which are in any way connected with dental science in any department, as of the greatest consequence Taken in an isolated state each new fact may be of but little practical value but taken as they are, linked in as a whole, and vast results flow from them. The first effort at the preparation of gold in a sponge state for the filling of teeth, did not prove satisfactory, but it has led to investigation and experiment, and we have now this valuable metal in a state of crystalization, perhaps about as soft and pliable as sponge, and yet when worked apparently as hard as foil.

In this Crystal Gold we believe that progress is being made as it regards tenacity and adaptation for the purposes of filling teeth; within the last three months we have noticed this improvement, as we think, in that manufactured by H. R. White & Co., Utica. N. Y., and more especially in some recently procured of Drs. Taft & Watt, of Xenia, Ohio, whose advertisement will be found in the present number of the Register. The Crystals of this are of a very fine quality, and there is less disposition to crumble or separate into small particles in handling. It compacts with great ease, and by some labor can be made as hard as any we have ever worked, and it bears a very fine finish.

There are two or three questions which may be asked in reference to the durability of this article when introduced as a filling.

Will it adapt itself to the cavity so as to be held as well as foil? From our observation, we think there can be no doubt of this. We would indeed suppose that the adaptation with the same amount of labor would be more perfect.

Will it retain its compactness and resist the permeation of moisture as well as foil? We confess this has been our only doubt as it regards its permanent utility, but, as yet, we see no evidence to support this supposition, and we are assured by Drs. Taft & Watt, who have been using it for a year past, almost exclusively, that it remains in every respect as perfect as the foil fillings.

Is it easy of introduction, and how will its cost compare with foil? We think it generally more easily introduced than foil, and yet we fear that in this one thing (its easy introduction) the greatest objection lies. We are satisfied that, although so easily introduced, yet great care is necessary to compact thoroughly and condense the entire filling. We thus far fill with it much as we do with foil, using small lumps somewhat in size and like our foil blocks, and introduce and compact in the same way, only using a larger pointed and roughened compacting instrument, and we find that the more labor we put on, the harder it becomes and the finer polish it will receive. After it is introduced, a few passes of a smooth instrument over the surface of the plug appears to almost finish the operation. This, however, is deceptive, for it still requires condensing, filing, stoning and polishing. As it regards cheapness, we do not think that an ounce of the Crystals will fill as many cavities of the same size as the same amount of foil. This may depend on two causes—first, more gold by weight may be put in each cavity, and second, the waste is, we think, greater. Continued use and care in manipulation may overcome this last, and the former cannot be considered an objection. We can only say to the Profession, try it carefully, and let not the idea of having a material so easily adapted to the cavity, prevent its proper and thorough preparation, and then labor and manipulate on each filling as long as if it was foil, and we think satisfaction will be the result.

We have long had faith in a selicious compound for filling teeth, which would take the place of gold. This, in composition and finish to resemble the tooth itself, has been our *beau ideal* for the perfection of this department of dental science. Our faith still wavers not, and if chemical science has so near developed this result from the metalic kingdom, why not perfect it from the mineral?

